# Influence of Denture Cleansers on the Retention Loss of Attachment Systems Retained Implant Overdenture

**DOI:** 10.1155/2023/5077785

**Published:** 2023-04-29

**Authors:** Hamad S. AlRumaih, Alhanouf A. Albarrak, Muneera AlMedaires, Ahmed A. Alsulaiman, Nadim Z. Baba, Faris A. Alshahrani, Firas K. Alqarawi, Yousif A. Al-Dulaijan, Fawaz Alzoubi, Abdulkareem A. Alhumaidan, Mohammed M. Gad

**Affiliations:** ^1^Department of Substitutive Dental Sciences, College of Dentistry, Imam Abdulrahman Bin Faisal University, P.O. Box 1982, Dammam 31441, Saudi Arabia; ^2^Department of Restorative Dental Sciences, College of Dentistry, Imam Abdulrahman Bin Faisal University, P.O. Box 1982, Dammam 31441, Saudi Arabia; ^3^Department of Preventive Dental Sciences, College of Dentistry, Imam Abdulrahman Bin Faisal University, P.O. Box 1982, Dammam 31441, Saudi Arabia; ^4^Advanced Dental Education Program in Implant Dentistry, Loma Linda University, School of Dentistry, Loma Linda, CL, USA; ^5^Department of General Dental Practice, Faculty of Dentistry, Kuwait University, P.O. Box 24923, Safat 13110, Kuwait

## Abstract

**Background:**

This study aimed to evaluate the effects of different denture cleansing solutions (DCSs) on the retention of Locator and Locator R-Tx attachment systems of implant retained overdentures (IRO).

**Methods:**

Two part acrylic resin blocks were fabricated, upper part contained metal housing and plastic inserts and lower part contained implant analogs and abutments. Eighty pink plastic inserts (40/attachment, 10/solution) were immersed in Corega, Fittydent, sodium hypochlorite, and water for a time simulating upto 1-year of clinical usage. Acrylic blocks were held on a universal testing machine for a pull-out test to record the dislodgement force. Measurements were conducted after 6 months (T1) and 12 months (T2). One-way ANOVA followed by Tukey's HSD test was used to analyze the results (*α* = 0.05).

**Results:**

For both attachments, retention significantly decreased after immersion in different solutions at T2 (*P* < 0.001). Locator R-Tx attachment in NaOCl showed a significant decrease in retention compared with other solutions at T1. At T2, there was a significant decrease in retention for all DCS compared with water (*P* < 0.001). Locator R-TX showed higher retention values per solution compared to Locator attachment (*P* < 0.001). In terms of retention loss %, NaOCl recorded the highest (61.87%) loss, followed by Corega (55.54%) and Fittydent (43.13%), whereas water demonstrated the best retention (16.13%) in both groups.

**Conclusion:**

Locator R-TX has better retention with different DCS immersion. The loss of retention varied with different types of DCS and NaOCl recorded the highest retention loss. Therefore, denture cleanser selection must be guided by the type of IRO attachment.

## 1. Introduction

Implant-retained overdentures (IROs) are an effective treatment option for edentulous patients [1]. IRO was developed to improve overdenture retention, stability, support, and chewing ability, as well as to preserve the surrounding bone and enhance patient satisfaction by utilizing various retentive attachment systems [2, 3]. Attachment selection is guided by the amount of retention needed, jaw anatomy, complexity of the case, implant alignment, vertical and horizontal prosthetic space, and patient compliance for recall appointments in order to perform adequate maintenance [3].

The most common attachment system in dental practice is the Locator® attachment system (Zest Anchors, Inc., Escondido, CA), which is a stud-type attachment and was designed to make restorative treatment with implant-supported overdentures easier [4, 5].

Compared with the ball and bar attachments, this system is relatively simple to fabricate and has shown clinically superior results in terms of postplacement prosthodontic complications and hygiene [6, 7]. Furthermore, Locator attachments have resiliency that helps distribute loads more evenly between the supporting dental implants and denture-bearing surfaces. These attachments have grown in popularity since their inception in 2001 owing to their ease of use and modest space requirements, as indicated by clinical reports of their use in situations where prosthesis space was restricted [6, 7].

Locator attachments can be used to replace existing ball abutments, particularly in patients who experience rapid wear of ball abutment components [8]. Moreover, these attachments have a rotational pivoting design, with the male component providing a resilient connection to the denture without retention loss. The full rotational movement of the denture cap over the male component enhances retention. Other factors contributing to the popularity of Locator attachments include excellent retention, small device dimensions (especially height), and durability of the components [9, 10].

Among all available stud systems, locators have the lowest profile, with a height of 2.5 mm [5, 11]. A titanium nitride coated cylindrical abutment with internal and external undercuts makes up the Locator “female” matrix, while the “male” is composed of a metallic housing with interchangeable plastic patrices [12]. The plastic inserts come in a variety of colors that correspond to different retention values. According to the manufacturer, a 10° variation between the direction of insertion of the plastic insert and the central axis of the matrix abutment is acceptable. As a result, these are advocated in cases where the interimplant angles range from 0° to 20°. When interimplant angles surpass 20°, an “extended-range” type of insert is recommended [12]. The Locator attachment has a dual retention strategy that combines inner and outer frictional modes to produce three distinct retention strengths, depending on the color of the plastic insert [13]. The overdenture seating of Locator attachment has a limited pivoting capability and wide coronal geometry [14].

The Locator® R-TX abutment system incorporates various enhancements over the original Locator abutment, in addition to a design that aids patient care. This innovative design allows for mobility in all axes, minimizing the amount of load placed on the implant and supporting bone [14]. According to the manufacturer, the Locator R-TX has an improved angle correction and pivot function, and is, therefore, recommended for implant angular discrepancies of upto 60° (maximum 30° per implant) [14, 15]. This is a significant advancement over the previous Locator attachment system, which only allowed a 40° diversion between implants, when used with extended-range inserts. In case of misaligned implants, the pivoting capacity of Locator R-TX system helps prevent damage to the retention inserts [14]. Locator R-Tx has internal hex abutment with an initiative hex pressure, presenting significantly less space for food/plaque accumulation and the absence of a principal stud inside the retention plastic inserts. However, there is no consensus regarding their long-term performance [14].

IRO demands rigorous hygienic care; adequate denture hygiene can help in the prevention as well as treatment of oral infections in edentulous patients [16]. Although brushing has been deemed insufficient for thorough denture cleaning, it remains one of the most prevalent methods of denture cleaning [17]. Furthermore, the ability of elderly patients to manually clean their dentures is often compromised [18]. Therefore, commercial denture cleansing solutions (DCSs) are often recommended for effective denture care [19, 20]. However, denture cleansers can have adverse consequences on overdentures and might cause deterioration of the denture base material, bleaching of acrylic resin, corrosion of metal, and destruction of temporary and soft lining substances if used incorrectly [21–24].

A previous study reported that DCSs are effective in controlling *Candida* infection, plaque deposition, and staining of dentures [24]. DCS may have a negative impact on prosthetic components, leading to loss of retention [21]. Nguyen et al. studied the influence of five DCS on Locator attachment retention and found a significant reduction in retention with sodium hypochlorite (NaOCl) and Efferdent, while Listerine increased the retentive value [23]. Other materials such as sodium bicarbonate-sodium perborate tablets and gel containing sodium laureth sulfate increased the retention of attachments. However, there was no significant change in retention using sodium bicarbonate [19].

Only a few investigators have studied the effects of DCS on the retention of the Locator attachments in the past. Moreover, the effect of DCS on the newly introduced Locator R-TX has not yet been investigated. The aim of this study was to evaluate the effects of different DCS on the retention of two commercially available attachment systems. The null hypothesis of our study was that DCS does not affect the retention of Locator and Locator R-TX attachment systems.

## 2. Materials and Methods

### 2.1. Specimen Preparation

**Figure 1:**
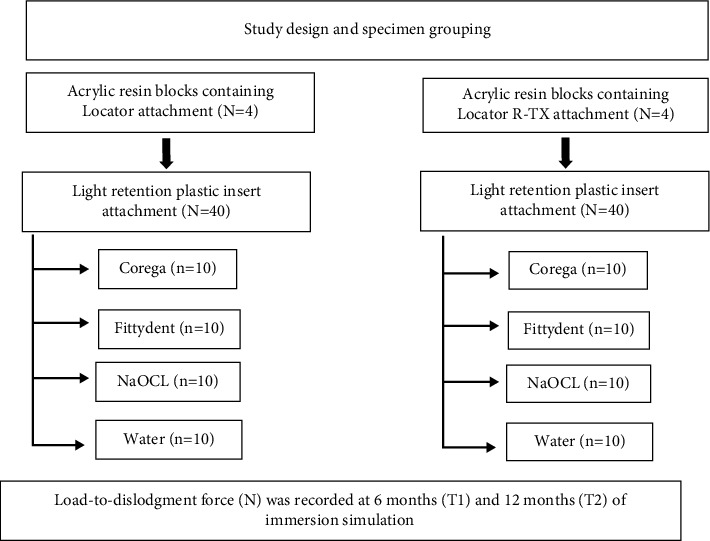
Flowchart of study design and specimen grouping.

The sample size was calculated using the G *∗* Power, version 3.0.10. (Franz Faul, Universitat Kiel, Germany) software. Using one-way ANOVA, a medium effect size of *f* = 0.4 (Cohen, J. 1988. Statistic power analysis in the behavioral sciences, Hillsdale, NJ: Erlbaum), 80% power, and at a 0.05 significance level (i.e., 95% confidence interval), the total required sample size of 10 specimens per group was needed to evaluate the effects of DCS on the retention of the Locator R-TX attachment in comparison with the Locator attachment system according to study design as shown in Figure 1.

**Figure 2:**
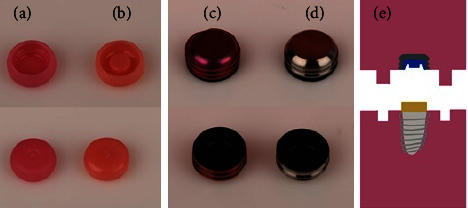
Rubber inserts (a and b) and metal housing (c and d); two acrylic blocks with metal housing containing rubber insert (e).

Figure 2 shows the rubber insert (a and b), metal housing (c and d), and two acrylic blocks (e). To examine the retention of the two systems, two acrylic blocks were prepared (one for each system). Each block was composed of two compartments. Upper compartment contained the metal housing, and lower compartment contained the implant analog of 3.7 mm diameter and abutments for each system (Zimmer Biomet, USA). These acrylic embedded implant analogs (one per attachment type) were immersed in three different DCS (Corega, Fittydent, and NaOCl; *n* = 10 for each subgroup) and tap water (control group) for varying time intervals to simulate 6 (T1) and 12 (T2) months of clinical usage (Table 1).

For preparing the acrylic blocks, a plastic mold (5 × 3.5 × 5 cm) with the requisite dimensions was produced, and molten wax was poured into it. Thereafter, a wax guide pin was used to position the implant analog in the middle of the wax block, perpendicular to the floor, with 3 mm of the implant analog visible above the wax surface using a dental surveyor. The wax block with the implant analog was flasked and treated using the traditional water bath method. The retrieved acrylic block was finished and tested for implant analog position after polymerization. In order to ensure an accurate fit of the upper compartment into the lower compartment, a groove of 4 mm (L) × 4 mm (W) × 3 mm (D) was made on both sides of the implant as an index to meet an opposing elevation in the metal housing block. The surface of the acrylic block was coated with Vaseline separating medium to create the acrylic component with the attachment. Molten wax was poured over the top of the acrylic block with the implant, using a plastic mold. This wax block was flasked and acrylized in the same manner as the preceding block.

The Locator and Locator R-TX abutments were then fitted into their respective implant analogs, followed by the metal housing. The metal housing was picked up by the upper block, which was attached with an autopolymerized acrylic resin. The upper and lower pieces were separated after polymerization and verified for a perfect fit, with no interference and positive fit indices.

### 2.2. Denture Cleanser Preparation and Immersion Protocol

The types and compositions of the denture cleansers used in this study are summarized in Table 1. Each attachment system was randomly separated into four groups based on the immersion solutions: Corega, Fittydent, NaOCl, and water as a control group (*n* = 10 each). According to the manufacturer's instructions, ten pink plastic inserts per denture cleanser solution were placed in perforated plastic bags and immersed in 125 ml of each cleanser, simulating 12 months of immersion (Table 1).

### 2.3. Testing Procedures, Data Collection, and Statistical Analysis

The load-to-dislodgment force (N) was recorded at 6 months (T1) and 12 months (T2) of immersion simulation. A Locator Core Tool was used to remove the processing inserts. Thereafter, Locator patrices were carefully placed into the metallic housings in the upper mounting using a Locator Core Tool with medium retention (pink) plastic inserts for each Locator type. The force experienced by the load cell during a single seating/unseating cycle was monitored to ensure proper attachment engagement. The load-to-dislodgment force was measured using an Instron universal testing machine (INSTRON 5965 US) with a 50 mm/min crosshead speed and a preset repeating insertion-removal cycle (N). All testing procedures were conducted and monitored by a single technical operator who was an expert in operating the equipment. Finally, data were collected, tabulated, computed, and statistically analyzed.

### 2.4. Statistical Analysis

Descriptive analyses were performed using means and standard deviations. Data were tested for normality using the Kolmogorov–Smirnov and Shapiro–Wilk tests. All values showed a parametric (i.e., normal) distribution. A paired *t*-test was performed to test for differences between water, Corega, Fittydent, and NaOCl across the two time points T1 and T2 in the Locator and Locator R-TX groups. Student's *t*-test was used to test for differences between Locator and Locator R-TX attachment groups at each time point separately (T1 and T2). ANOVA and Tukey's multiple comparison test were performed to test for differences between water, Corega, Fittydent, and NaOCl in the two study groups at 6 months and 12 months. Statistical significance was set at *P* < 0.05. All statistical analyses were performed using SAS statistical software package, version 9.4 (SAS Institute Inc., Cary, NC, USA).

## 3. Results

**Figure 3:**
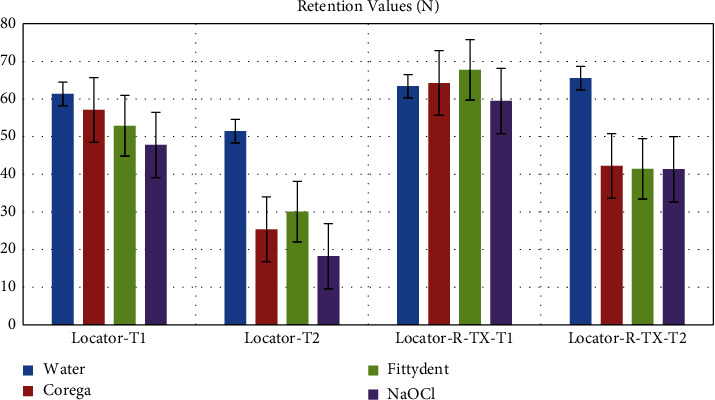
Mean values of retention of the two Locator systems with respect to the cleansing solutions at the two test time points.

The means, standard deviations, and statistical significances of retention for the two types of Locator attachments for each DCS are summarized in Table 2 and Figure 3. The results of ANOVA revealed a significant decrease in retention after soaking both Locator types in the different cleansing solutions (*P* < 0.001), except for Locator R-TX immersed in water (*P*=0.474). With respect to the multiple comparisons (Tukey post hoc test) of different DCS at T1 and T2 for the two types of Locator attachments, there were significant differences in retention between all cleansing solutions at T1 and T2 (*P* < 0.001). The water groups showed the highest retentive values at T1 (61.34 ± 4.44 N) and T2 (51.45 ± 3.27 N), while the NaOCl groups showed the lowest retentive values at T1 (47.78 ± 1.44) and T2 (18.22 ± 1.72). In terms of retention loss %, NaOCl recorded the highest (61.87%) loss, followed by Corega (55.54%) and Fittydent (43.13%), whereas water demonstrated the best retention (16.13%) in both groups.

According to Tukey's post hoc test results (Tables 3 and 4), Locator R-TX showed a significant decrease in retention in NaOCl compared to water, Corega, and Fittydent at T1, while there was no significant difference in retention of water vs. Corega and Corega vs. Fittydent at T2. Overall, there was a significant decrease in retention in all DCS compared to water (*P* < 0.001). No significant differences of retention were observed between Corega and Fittydent or Corega and NaOCl. In term of retention, Fittydent recorded the lowest values (38.86%), followed by Corega (34.28%) and NaOCl (30.5%), and water showed the best retention among all groups (−3.39%).

**Figure 4:**
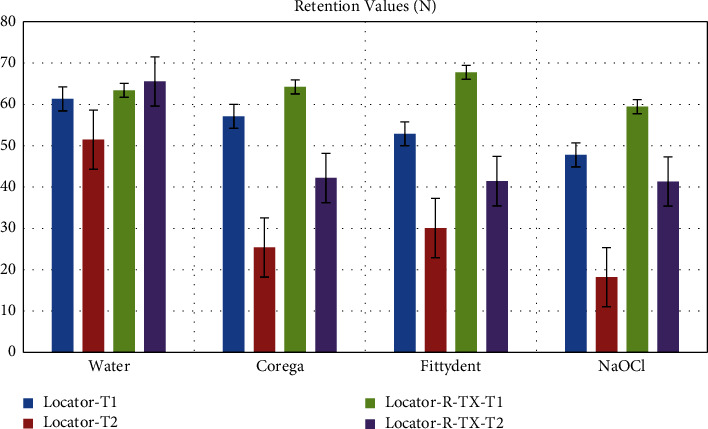
Comparison of retention values between both attachments in concerned solution as an effect of time.

Upon intergroup comparison between the Locators (Table 5 and Figure 4), water immersion at T1 showed no significant differences between the two attachment groups (*P*=0.266), while Locator R-TX showed significantly greater retention when compared with the Locator system for all solutions at both recall times (*P* < 0.001).

## 4. Discussion

IRO requires meticulous hygiene maintenance for improved lifespan and durability. Denture wearers are instructed to follow home care measures in order to maintain healthy oral mucosa. The method of hygiene may affect the overdenture attachment and retention. This study tested the effect of different DCS on the retention of the Locator and Locator R-TX attachment systems after a simulated period of upto 1 year of clinical usage [25, 26].

In this study, two types of attachments were evaluated using four types of DCS. The effect of DCS on the Locator R-TX attachments of the IRO has not been investigated to date. Our results showed that when selecting a cleaning solution, it is important to consider which cleaning solution will result in the best long-term retention when used with a specific attachment system. For removable dental prostheses wearers, different denture cleansers and cleaning methods were recommended to prevent microbial adhesion and subsequently prevent denture stomatitis occurrences [19, 27]. Effervescent denture cleansers (Corega and Fittydent) are most commonly used due to their efficacy in decreasing *C. albicans* adhesion in addition to its ability in removing light stains and debris loosing from denture surfaces [19, 20]. On the other hand, NaOCl is one of the most common used disinfectant solutions with its effectiveness in reducing *C. albicans* adhesion due to its fungicidal properties [19]. Due to the different compositions and immersion times of denture cleansers, the clinical use might affect the properties of denture base resin as well as attachment systems; therefore, these representative cleansing solutions were selected in the present study.

The null hypothesis that DCS will not affect the retention of the attachment systems was rejected. After a simulated period of 12 months, a significant decrease was observed in the peak load-to-dislodgement force of both attachment systems. It was observed that the loss of retention values in Locator attachments immersed in NaOCl solution was much higher than that in the other cleansers at all time intervals, which is consistent with the findings of multiple previous studies [22, 23, 28]. It was previously reported by Evtimovska et al. that cleaning IRO with NaOCl significantly decreased the retentive values of the Locator attachments [29]. According to Kürkcüoğlu et al. [28], NaOCl altered the surface morphology of the plastic attachments, resulting in porosities and cracks. However, no significant difference was found in the effect of water and NaOCl on the retentive value of pink plastic inserts [28]. Furthermore, in our study, when all cleansing solutions were compared with water, Corega came in second place after NaOCl in terms of considerably lowering the retention values of Locator attachments at all intervals.

The data obtained from this study demonstrate that water affects the retention of Locator attachments, but has no impact of the retention of Locator R-TX attachments. This may be attributed to the differences in design between the two attachments. Further studies are needed to confirm the direct relationship between the denture attachment design and retention [30]. Hypochlorite solutions have been shown to exhibit bactericidal and fungicidal effects on the organic constituents of the plaque matrix. Because of its bleaching and deteriorating effects on acrylic resins, NaOCl may be the most effective denture immersion solution with antifungal activities in the market [5, 18]. However, the soaking time should not exceed 10 minutes [18]. Due to lack of literature on the Locator R-TX attachment, the results of the current study should be interpreted with caution until further investigations confirm our findings.

It has been reported that patients with IRO remove their prostheses at an estimated speed of 50 mm/min; therefore, the same speed was selected for the pull-out test in the current study [2, 23, 28]. In addition, a one-pull test was performed based on the recommendations of previous studies [23, 29, 31], which revealed that a significant loss of retention occurs after the first separation of attachments and abutments. The simulation immersion times in our study were selected based on previous studies conducted to test the retention values of IRO [2, 23, 28].

According to the findings of the present study, frequent replacement of attachments is crucial when using NaOCl as a cleansing agent. Since NaOCl was observed to gradually reduce the retention of Locator attachments, it should not be used as a regular denture cleanser. This is the primitive study on R-TX so the results should be explained with cautions to implement clinically. Due to the variations between effects of denture cleanses with time (long-term use), the type of cleanser is considered regardless the attachment type. Additionally, the resin surface properties must considered as the denture cleansers affected the resin surface properties and has adverse effects on the color and esthetics [32]. This is considered a point for further studies with long durations and different denture cleansers. When more findings are reported in future studies, this will be more beneficial in usage of rubber insert for long time with appropriate amount of retentions and decrease the need for periodic rubber inserts replacement.

This in vitro study had some limitations. First, the plastic inserts were immersed in the cleansers for a maximum of 12 months simulation period, although longer immersion times might result in more changes. Second, the attachments were not tested in a realistic oral environment or with natural occlusal forces. This may have concealed certain effects of the denture cleansers on the attachments. Therefore, further in vivo studies are recommended to evaluate the clinical retention loss different attachment systems after using different denture cleansers.

## 5. Conclusions

Within the limitations of present study, the following conclusion could be drawn:

1. The retention of the Locator as well as Locator R-TX attachment systems decreased with immersion in all types of DCS.
2. R-TX attachments showed higher retention values compared to the Locator attachments with all types of DCS.
3. The lowest retention values for both attachments were recorded after immersion in NaOCl.
4. Due to the varying effects of DCS on the different attachment systems, the selection of denture cleanser should be based on the clinician's recommendation. In addition, periodic replacement of that the plastic inserts is recommended to improve long-term denture retention.

## Figures and Tables

**Table 1:** Denture cleaning solution preparation and immersion protocol.

Solution/manufacturer	Composition	Preparation/temperature	Time/day	Total immersion time (12 months)
Water	—	—	8 hours	2880 hours
				6 months; 1440 h
				12 months; 2880 h
Corega	Potassium monopersulfate; sodium bicarbonate; sodium lauryl sulfoacetate; sodium perborate monohydrate; sodium polyphosphate	One tablet dissolved in 200 mL of warm tap water (40°C)	3 minutes	(18 hours)
				6 months; 9 h
				12 months; 18 h
Fittydent	Sodium perborate, sodium bicarbonate, potassium monopersulfate, trisodium phosphate, PEG-240, sulfamic acid, PVP, TAED, silica, sodium methyl oleoyl taurate, cellulose-lactose, color C.I. 42090, aroma	One tablet dissolved in 200 mL of warm tap water (40°C)	5 minutes	(30 hours)
				6 months; 15 h
				12 months; 30 h
NaOCI	5.25% NaOCl (1 : 1 ratio of sodium and chloride ions) diluted 1 : 10 in tap water	Solution of 5.25% sodium hypochlorite (NaOCl, 1 : 5 dilution) was diluted to obtain 1% NaOCl solution by adding 50 mL NaOCl to 200 mL water	10 minutes	(60 hours)
				6 months; 30 h
				12 months; 60 h

**Table 2:** Means, SDs, and statistical significances of retention of both Locator attachment systems in different cleaning solutions at the two time points.

Attachment system	Solutions	T1 mean (SD)	T2 mean (SD)	T2−T1 mean difference (SD)	Retention loss in %	*P* value
Locator attachment	Water	61.34 (4.44)	51.45 (3.27)	−9.89 (4.93)	16.12%	0.0001
	Corega	57.08 (1.09)	25.38 (0.65)	−31.70 (1.30)	45.84%	<0.0001
	Fittydent	52.89 (2.36)	30.08 (1.31)	−22.82 (2.42)	43.14%	<0.0001
	NaOCI	47.78 (1.44)	18.22 (1.72)	−29.56 (1.98)	61.75%	<0.0001

Locator R-TX attachment	Water	63.39 (3.49)	65.54 (7.31)	2.15 (9.08)	−03.93%	0.474
	Corega	64.22 (4.56)	42.20 (0.90)	−21.50 (5.35)	33.47%	<0.0001
	Fittydent	67.74 (1.80)	41.42 (2.25)	−26.32 (3.21)	38.85%	<0.0001
	NaOCI	59.48 (0.88)	41.34 (1.86)	−18.15 (1.82)	30.51%	<0.0001

SD: standard deviation. *P* value <0.05 is significant.

**Table 3:** Pairwise comparisons of the cleaning solutions in the Locator group.

Recall times	*F*-value	*P* value	Multiple comparison Tukey's test					
			Water vs. corega	Water vs. fittydent	Water vs. NaOCI	Corega vs. fittydent	Corega vs. NaOCI	Fittydent vs. NaOCI
6 month	47.16	<0.0001	0.0055	0.0010	0.0010	0.0066	0.0010	0.0010
12 months	519.32	<0.0001	0.0010	0.0010	0.0010	0.0010	0.0010	0.0010

*P* value <0.05 is significant.

**Table 4:** Pairwise comparisons of the cleaning solutions in the Locator R-TX group.

Recall times	*F*-value	*P* value	Multiple comparison Tukey's test					
			Water vs. corega	Water vs. fittydent	Water vs. NaOCI	Corega vs. fittydent	Corega vs. NaOCI	Fittydent vs. NaOCI
6 month	12.44	<0.0001	0.8999	0.0147	0.0324	0.0633	0.0069	0.0010
12 months	84.84	<0.0001	0.0010	0.0010	0.0010	0.8999	0.8999	0.8999

*P* value <0.05 is significant.a

**Table 5:** Mean values, SDs, and significance of retention loss of the two Locator systems in different solutions at the defined time points.

Recall time	Variable	Locator mean (SD)	Locator R-TX mean (SD)	Locator (R-TX)−locator mean difference (SD)	*P* value
T1	Water	61.34 (4.44)	63.39 (3.49)	2.05 (1.79)	0.2669
	Corega	57.08 (1.09)	64.22 (4.56)	7.14 (1.48)	0.0007
	Fittydent	52.89 (2.36)	67.74 (1.80)	14.85 (0.93)	<0.0001
	NaOCI	47.78 (1.44)	59.48 (0.88)	11.70 (0.534)	<0.0001

T2	Water	51.45 (3.27)	65.54 (7.31)	14.09 (2.53)	<0.0001
	Corega	25.38 (0.65)	42.20 (0.90)	16.82 (0.35)	<0.0001
	Fittydent	30.08 (1.31)	41.42 (2.25)	11.34 (0.82)	<0.0001
	NaOCI	18.22 (1.72)	41.34 (1.86)	32.12 (0.80)	<0.0001

*P* value <0.05 is significant.

## Data Availability

The data used to support the findings of this study are available from the corresponding author upon request.
